# Kill them with kindness: preliminary evidence for women’s competitive altruism

**DOI:** 10.3389/fpsyg.2026.1924479

**Published:** 2026-09-09

**Authors:** Maryanne L. Fisher, Oriana Figueroa, Nohelia Valenzuela, Ferity Thorne, Pablo Polo, José Antonio Muñoz-Reyes, Nerea Aldunate, Simón Ramirez

**Affiliations:** 1 Department of Psychology, Saint Mary’s University, Halifax, NS, Canada; 2 Núcleo de Investigación en Interacciones Sociales (NIIS), Laboratorio de Fisiología y Comportamiento Humano (LABFICH), Facultad de Salud y Ciencias Sociales, Escuela de Psicología, Universidad de las Américas, Santiago, Chile; 3 Laboratorio de Comportamiento Animal y Humano, Centro de Investigación en Complejidad Social, Facultad de Gobierno, Universidad del Desarrollo, Santiago, Chile

**Keywords:** competition, competitive altruism, prestige, reputation, women

## Abstract

**Introduction:**

We developed the Competitive Altruism Survey (CAS) to test how intrasexual competition (i.e., competitive altruism) relates to Machiavellianism, Big Five personality traits, social desirability, and impression management.

**Methods:**

A sample of 103 heterosexual cisgender women aged 18 to 45 years completed an online survey. Results: Competitive altruism was strongly and positively related to Machiavellianism and negatively related to Agreeableness and was more used when directed at other women than at men. Neuroticism was correlated with anxiety and frustration that accompany strategic kindness, rather than to competitive altruism. Correlations with impression management and social desirability were unexpectedly negative; this pattern is open to several readings, one being openness about strategic intent. Open-ended responses converge with this interpretation, with women describing the strategic kindness they observe in others as occurring within workplace hierarchies, in publicized or social-media display, and in warmth that contradicts itself in private.

**Discussion:**

Our results offer preliminary self-report evidence, in a predominantly Western online sample, that women may use competitive altruism and generosity as strategies to gain a reputation in competition.

## Introduction

Human generosity toward non-kin has long posed a puzzle for evolutionary perspectives of behavior, particularly when helping carries a cost and yields no direct benefit (Fehr and Fischbacher, 2003; Nowak, 2006; Trivers, 1971; West et al., 2007). However, helping behavior, kindness, and generosity may serve as a form of advertisement of one’s qualities. Building on Zahavi’s (1975) argument that costly traits can act as honest signals of gene quality, Roberts (1998) proposed that altruism functions as such a signal whenever individuals compete to be chosen as partners, a process he termed competitive altruism. Where desirable partners are scarce and choosy, it is advantageous to appear more willing and able to help than one’s rivals, and the resulting reputation attracts cooperative partners (Barclay, 2013; Barclay and Willer, 2007; Van Vugt et al., 2007). Indeed, experimental findings show people behave more generously when their contributions are made public and with the most generous group members acquiring status and being preferred as future partners (Hardy and Van Vugt, 2006).

Much of the experimental evidence for competitive altruism has been focused on men, and from public, conspicuous forms of altruism. That which does address women provides mixed evidence, or shows it is less consistent than in men, with most work mainly focused on non-mating contexts (e.g., Böhm and Regner, 2013; Giardini et al., 2021; Sylwester and Roberts, 2010; Tognetti et al., 2016). Men become more generous in the presence of a potential mate (Iredale et al., 2008, 2020; Webb and Fisher, 2018), donate competitively to attractive female fundraisers once a rival has donated (Raihani and Smith, 2015), and compete to occupy self-sacrificial heroic roles more readily than women (McAndrew and Perilloux, 2012). Across these studies, generosity (and perhaps by association, kindness) appears as a conspicuous, visible, costly, and often mating-directed display, performed for an audience and aimed at advertising oneself to prospective mates, a type of competition that is less characteristic of women.

Women are far from indifferent to competition, though their rivalry tends to be conducted at low physical risk and through indirect means. Campbell (1999) argued that selection favored a lower threshold for fear in women because a mother’s survival mattered disproportionately for her offspring’s survival. Consequently, open, escalating contests are more common among men, and women’s competition tends toward forms that are less physically harmful (but see Fisher, 2026). Consistent with that view, women rely heavily on indirect aggression such as gossip, social exclusion, and the derogation of a rival’s appearance and reputation (Vaillancourt, 2013; Fisher, 2017), tactics that damage a competitor while shielding the aggressor from retaliation. These indirect tactics may be closer to scramble competition, in which a resource is divided among all who reach it, than to contest competition, in which access is settled by direct interference between winners and losers (Benenson and Abadzi, 2020). Women competing under such a strategy reach dispersed resources first and build the alliances and support that secure continued access, while avoiding the relational and physical costs of confrontation. Derogation, gossip, and exclusion are low-cost interference tactics and reduce a rival’s standing without an overt contest. Reynolds et al. (2025) extended this possibility, finding that gossip phrased as concern for its target carries a reputational cost for the target and none for the gossiper. Girls and women also manage their competition through the careful maintenance of alliances and the quiet policing of other women, competing openly only from positions of relative safety (Benenson, 2013). The same preference for subtle instead of overt tactics appears in research on mate competition, where self-promotion and the quiet manipulation of rivals occur concurrently with more direct derogation (Fisher and Cox, 2011). Prosociality and competitiveness also become linked in women under particular social conditions. Women are more likely to enter competition when winning includes the option to share part of the reward with losers (Cassar and Rigdon, 2021), and the sex difference in competitiveness disappears when rewards can benefit offspring (Cassar et al., 2016). Neither finding demonstrates competitive altruism in the strict sense of visible rivalry over who gives more but instead that women enter competition more readily once winning carries some benefit for others.

If competitive altruism rewards those who appear unusually willing to help, and if women’s competition often favors tactics that are low in risk and easily disguised as something other than competition, then kindness may be an ideal competitive tool for women. Kindness is socially rewarded (e.g., Rucas et al., 2006), and it is difficult to construe as an attack while it wins trust and allies. Thus, we propose that some of women’s prosocial behavior is a low-risk competitive tactic in which kindness secures allies and reputational advantage while remaining deniable as competition. The familiar expression ‘kill them with kindness’ points to this possibility. Women’s competitive altruism may be less a public contest over who gives more, such as observed among men, and instead a matter of defending reputation among other women, where generous or helpful behavior buys standing and some protection from exclusion.

Treating kindness as a possible competitive strategy leads to the question of whether the women whose prosociality is most strategic can be distinguished from those whose kindness is more sincere and genuinely directed toward the well-being of others. A strategic use of kindness should be indicative of a manipulative orientation; hence, we hypothesized (Hypothesis 1a) that competitive altruism is positively associated with Machiavellianism, the disposition to treat social life as something to be managed for personal advantage (Christie and Geis, 1970). Further, while some individuals may rely on kindness as a manipulative tool, others may use it in a publicly visible way, or to seek admiration. Thus, we further hypothesized (Hypothesis 1b) that the relationship with competitive altruism would be observed primarily for manipulation-based survey items, rather than evenly across all items. Machiavellianism may also influence how women perceive others, and we hypothesized that women high in Machiavellianism would more readily attribute strategic kindness to other women (Hypothesis 1c).

For the same reason, we hypothesized that competitive altruism would be independent of the genuinely warm and trusting facets of personality, such that Agreeableness would show a weak, nonsignificant relationship with competitive altruism overall, and a negative association with the manipulation items of the scale (Hypothesis 2a). Other Big Five personality dimensions could be important, too. Extraversion is characterized by a tendency to seek and to receive reward from social attention (Ashton et al., 2002), and visible acts of generosity attract notice and confer status (Hardy and Van Vugt, 2006). As a result, we hypothesized that Extraversion would correlate positively with competitive altruism, particularly with the items capturing publicly visible behavior (Hypothesis 2b). Conscientiousness, with its emphasis on self-regulation, deliberate goal pursuit, and adherence to social norms, has been linked to prosocial behavior such as following through on commitments and to acting in socially expected ways (Habashi et al., 2016). We argue that strategic kindness is sustained, deliberate, and reputation-oriented rather than impulsive, and consequently, we hypothesized that Conscientiousness would correlate positively with competitive altruism (Hypothesis 2c). Neuroticism, the disposition toward negative affect, vigilance for negative evaluation, and rumination (Soto and John, 2017), should express itself less in the strategic deployment of kindness and more in the worries and frustrations that accompany kindness when it is used strategically. The concern that one’s generosity might be perceived as insincere, and the frustration of having generous acts go unnoticed, are negative emotional reactions to which more neurotic women may be more susceptible. Thus, we hypothesized that Neuroticism would be positively correlated with anxiety and frustration (in a competitive altruistic context), and not with competitive altruism generally (Hypothesis 2d). Openness, capturing curiosity, imagination, and aesthetic sensibility, has no clear theoretical link to the strategic use of kindness as a competitive tool, and we made no directional prediction for its relationship with competitive altruism (Hypothesis 2e).

A woman who uses kindness strategically may also try to present herself in an especially favorable light when reporting on her own behavior, since both competitive altruism and the management of how one is perceived are tied to social standing. Impression management captures the conscious effort to claim virtuous behaviors and to deny socially undesirable ones (Leary and Kowalski, 1990; Paulhus, 1984), while social desirability is the tendency to seek approval and avoid disapproval through self-presentation (Crowne and Marlowe, 1960). Strategic kindness could exploit these; hence, we hypothesized that competitive altruism would correlate positively with both impression management and social desirability (Hypothesis 3a). Any link between impression management and competitive altruism should therefore appear in the items describing publicly visible generosity and not in those describing more private or manipulative uses of kindness. We therefore hypothesized (Hypothesis 3b) that impression management will be most observed for survey items related to public display than by the rest. Similarly, social desirability reflects a need for approval, and we expect it to correlate with items about seeking admiration and the frustration of having generous acts go unnoticed. We hypothesized (Hypothesis 3c) that the association with social desirability would be strongest for the admiration and frustration items of the scale.

Last, we hypothesized (Hypothesis 4) that the audience of women’s behavior matters. Women’s competition is conducted mainly with other women rather than with men (Benenson, 2013; Campbell, 1999; Vaillancourt, 2013), and the reputation, alliances, and everyday comparisons of kindness and standing that motivate intrasexual competition operate most consequentially within the female group. Beyond reputational stakes, women may have an insider’s understanding of when kindness is being used as a strategic move, both because they spend more of their daily time in the company of other women than of men (e.g., David-Barrett et al., 2015) and because they may have deployed such warmth themselves. Thus, competitive altruism should be more readily seen in behaviors directed at other women than in behaviors directed at men.

We do not argue that women’s kindness is generally insincere, or reduce prosociality to strategy, given, at least at the mechanistic level, genuine altruism exists (Batson, 2011). Our concern is whether a measurable part of that kindness is strategic. Therefore, we examine competitive altruism in relation to Machiavellianism, agreeableness, social desirability, and impression management, and we asked whether strategic kindness is directed differently toward women and toward men, given that women’s competition is typically aimed at other women.

## Method

### Participants

Participants (*n* = 113) were recruited through the online research platform Prolific and financially compensated for their time. All participants were screened by Prolific for their ability to read and write in English. Eligibility was restricted to cisgender women because the competitive-altruism framing concerns same-sex and mate-relevant contexts. For that reason, the analytic sample was further restricted to women who confirmed via Prolific screening and again on the survey that their sexual orientation is heterosexual. Eight respondents failed one or more of the three attention checks (i.e., an open-ended response following a visual search task, similar to an I-Spy game), and a further two respondents who did not describe themselves as heterosexual were excluded, yielding a final analytic sample of 103 heterosexual cisgender women. The restriction to heterosexual participants was not preregistered; a sensitivity analysis re-including these two respondents is reported in the Results.

The women ranged in age from 18 to 45 years (*M* = 33.7, *SD* = 6.6). Most lived in the United Kingdom (37%), the United States (31%), or Canada (26%), with the remaining (6%) in Sweden or South Africa. Sixty-two percent described their ethnic or cultural background as white or European, 17% as Black or African, 6% as Asian, and 15% as Hispanic, mixed, or of other heritage. Sixty-five (63%) held a university bachelor’s degree or higher. Seventy-seven (75%) were in a current romantic relationship, with 46 (45%) married or in a common-law partnership and 31 (30%) in an exclusive partnership; 22 (21%) were single, 2 (2%) were separated, 1 (1%) divorced, and 1 (1%) described her status as complicated or other. Forty-four (43%) reported having children.

### Measures

This study was pre-registered at https://osf.io/78pve/registrations and reviewed by institutional Research Ethics Boards. All constructs were assessed by self-report within a single online survey. Every multi-item scale was scored following its published key, with reverse-keyed items recoded before averaging.

#### Competitive altruism

Competitive altruism was assessed with a newly developed Competitive Altruism Survey (CAS; see Appendix A), comprised of 15 statements that describe the strategic use of kindness and generosity. These items were based on a review of recent academic literature (e.g., Reynolds et al., 2025), as well as unpublished qualitative data from women about dating competition (aggregate qualitative data reported in Fisher and Cox, 2011). Each statement was rated on a five-point Likert-type scale ranging from 1 (strongly disagree) to 5 (strongly agree), and each was presented twice, once with respect to other women and once with respect to men, producing 30 ratings in total. A competitive altruism total was calculated across all 30 ratings (Cronbach’s *α* = 0.95), and women-directed (*α* = 0.91) and men-directed (*α* = 0.92) sub-scores were formed by averaging the 15 ratings within each audience.

The 30 ratings were submitted to a principal components analysis. No rotation was applied because only the first component was interpreted. The first component was dominant, accounting for 40.5% of the item variance, with an eigenvalue of 12.14 against 2.32 and 1.97 for the next two components. We recognize that a high Cronbach’s alpha does not establish unidimensionality; the presentation of each statement across two audiences creates dependence between the paired items, and a parallel analysis retained three components, each with an eigenvalue exceeding the mean of the corresponding eigenvalues from 1,000 random datasets. However, McDonald’s omega (ω) = 0.95 for the total (ω = 0.91 for the women audience; ω = 0.92 for the men audience sub-scores), the two audience composites correlated (*r* = 0.71), and all but one of the 30 ratings loaded at or above 0.40 on the first unrotated principal component. We, therefore, treat the scale as a preliminary, study-specific instrument whose structure requires confirmation in a larger, independent sample.

Several conceptually defined subsets of the items were examined. The manipulation, public-display, and admiration constructs were preregistered, each with example items and a stated rationale, and the audience subsets were preregistered in full. The complete assignment of the individual statements to the manipulation, public-display, and admiration subsets was finalized before analysis on that basis, rather than listed item by item in the preregistration. We treat these subsets as hypothesis-specific indices rather than validated subscales.

Every statement was rated once with women as the intended audience and again with men, meaning a grouping of seven statements per audience formed a 14-item composite. Further, a grouping of two formed a four-item composite, giving a manipulation subset (14 items, *α* = 0.91), a public-display subset (14 items, *α* = 0.92), and an admiration-seeking subset (4 items, *α* = 0.81). The subsets are not mutually exclusive, given two statements describe behavior that is instrumental and publicly performed and thus contribute to both the manipulation and public-display subsets (meaning combined counts exceed the 30 rated statements). A full key matching each statement to its subset appears in Appendix A.

#### Perceptions of competitive altruism

Two sets of 10 questions assessed perceptions of how other women use kindness and generosity strategically, one set about behavior directed at other women (Cronbach’s *α* = 0.89) and the other about behavior directed at men (*α* = 0.84). Both sets used the same 1 (strongly disagree) to 5 (strongly agree) scale as the CAS; see Appendix A.

#### Self-reflection items

Seven items asked about the respondent’s self-reflections on kindness and generosity, rated again on the 1 (strongly disagree) to 5 (strongly agree) scale. Two of these items were directed at genuine kindness, such as feeling good about helping when no one notices, and were retained as content checks and excluded from the competitive-altruism composites. Four of the remaining items, concerning feeling a need to prove one’s kindness to women and to men, and worry that the kindness might be perceived as insincere, were measured along with the two frustration items (I have felt frustrated when my acts of kindness or generosity went unnoticed by other women/men) from the CAS, creating a preregistered six-item anxiety-and-frustration composite (Cronbach’s *α* = 0.85). A seventh item asked participants whether they had felt competitive with other women in social or professional settings and was retained as a single-item index for exploratory analysis. See Appendix A.

#### Machiavellianism, social desirability, and impression management

Machiavellianism was measured with the nine-item Machiavellianism subscale of the Short Dark Triad (SD3; Jones and Paulhus, 2014), which captures a strategic and exploitative interpersonal orientation indicated by calculated self-interest, manipulation of others, and a willingness to deceive when doing so serves one’s ends. Items were rated on a five-point agreement scale from 1(strongly disagree) to 5 (strongly agree). The mean was calculated across the nine items, with higher scores indicating greater Machiavellian tendencies, and Cronbach’s *α* = 0.81.

General social desirability was measured with the 33-item Marlowe–Crowne Social Desirability Scale (Crowne and Marlowe, 1960), which addresses the tendency to portray oneself in line with prevailing social norms. Fifteen items were reversed prior to scoring, such that a value of 1, of the true-false responding options, indicated the socially desirable response. A total across the items created a score ranging from 0 to 33, with higher scores indicating a stronger general tendency toward socially desirable responding; Cronbach’s *α* = 0.84.

Impression management was measured with the 20-item Impression Management subscale of the Balanced Inventory of Desirable Responding (BIDR-IM; Paulhus, 1991). Items were rated on a scale from 1 (not true at all) to 5 (very true). The ten odd-numbered items were reverse-coded so that higher scores reflected a stronger tendency to claim implausibly virtuous behavior. The mean across the 20 items was calculated, with higher values indicating greater impression management; Cronbach’s *α* = 0.82.

#### Personality

The five personality domains were measured with the 60-item Big Five Inventory-2 (BFI-2; Soto and John, 2017), an updated version of the original Big Five Inventory that captures Extraversion, Agreeableness, Conscientiousness, Neuroticism, and Openness with 12 items per domain. The BFI-2 substitutes the labels Negative Emotionality for Neuroticism and Open-Mindedness for Openness, although we retain the conventional names here to ease comparison with prior research. Thirty items, six per domain, were reverse-coded, and a mean for each domain was calculated across its 12 items, with higher values indicating more of that trait. Internal consistency was Cronbach’s *α* of 0.87 for Extraversion, 0.85 for Agreeableness, 0.91 for Conscientiousness, 0.93 for Neuroticism, and 0.88 for Openness.

#### Self-rated traits

Self-rated trait adjectives were collected as an exploratory measure of how women high in competitive altruism describe themselves relative to other women. Thirteen adjectives were presented under the stem “Compared with other women who are around the same age as you, how would you rate yourself on the following characteristics?” and rated on a five-point scale with 1 = much less, 3 = about the same, and 5 = much more. The adjectives covered three loose conceptual clusters, namely warmth (Kind, Generous, Friendly), prestige and achievement (Intelligent, Successful, Popular, Funny, Wealthy, Ambitious), and a strategic-and-disagreeable cluster (Manipulative, Deceptive, Fake, Honest (reverse scored)). The framing of peers was used because it situates self-ratings in the same intrasexual context as the CAS, and absolute self-ratings on socially loaded adjectives such as ‘manipulative’ may suffer floor effects. The 13 items were treated as a single-item rating and correlated separately with the CAS composites.

### Procedure

The study was administered online through Qualtrics. After providing informed consent, participants completed the measures. The three attention-check items were embedded among the questionnaires. At the close of the survey, participants responded to an open-ended prompt asking them to describe a time when they had noticed a woman using kindness or generosity to gain status or to manipulate others. Participants were then debriefed.

### Statistical analysis

Directional hypotheses were tested with one-tailed tests and nondirectional hypotheses with two-tailed tests. Associations were estimated as Pearson correlations with 95% confidence intervals and were confirmed against a distribution-free Spearman coefficient, and Bayes factors were reported throughout so that evidence for a null could be weighed against evidence of an association. Where a registered prediction concerned the specificity of an association to a particular set of items, the difference between the two dependent correlations was tested using Williams’ (1959) procedure for dependent overlapping correlations. When a family of related predictions was examined, the false discovery rate was controlled with the Benjamini–Hochberg procedure (Benjamini and Hochberg, 1995). With *N* = 103, the smallest population correlation detectable at 80% power and α = 0.05 was approximately *r* = 0.27 for a two-tailed test and *r* = 0.24 for a one-tailed test.

## Results

**Figure 1:**
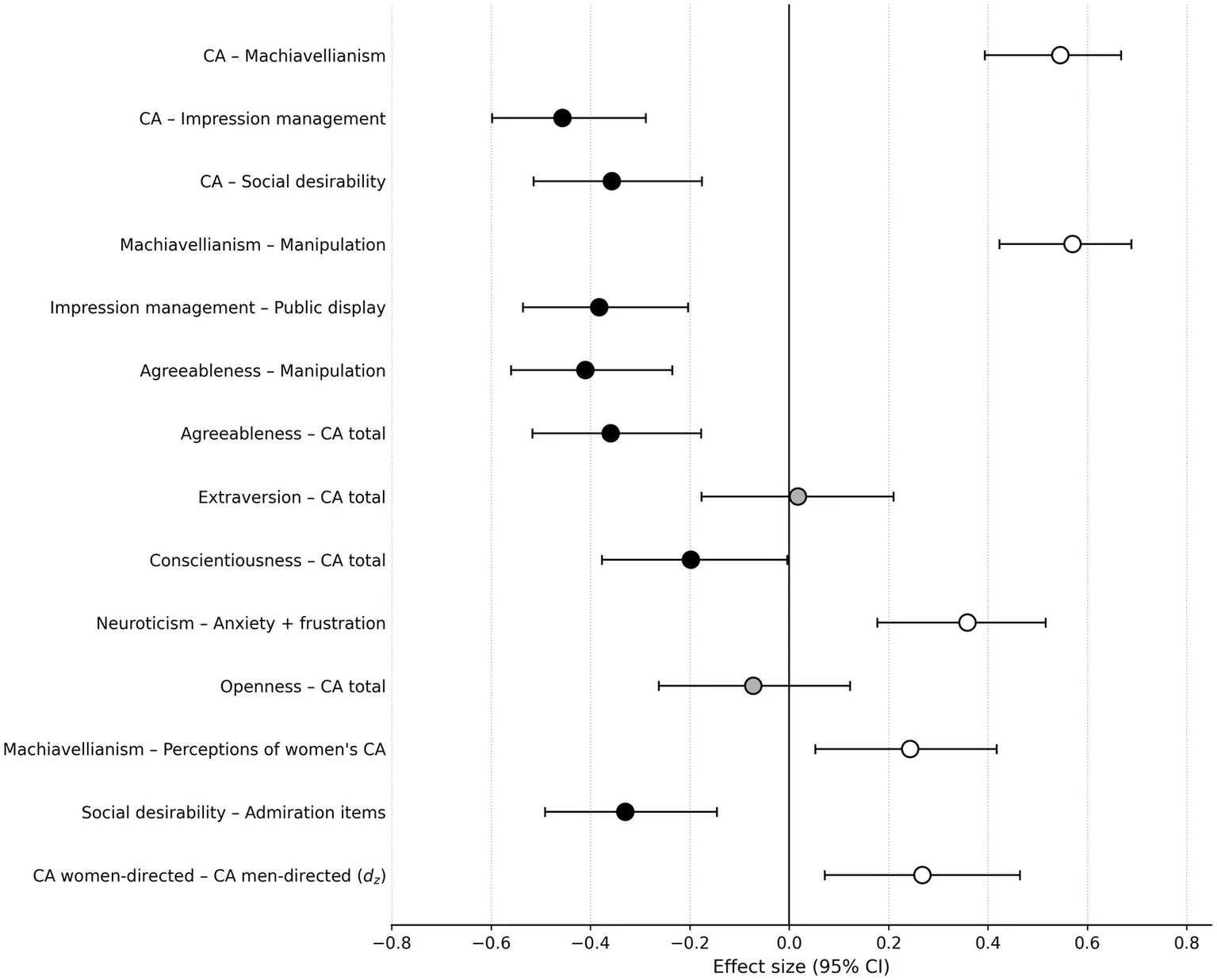
Correlations with competitive altruism and its composites (*N* = 103). Points are effect sizes and bars are 95% confidence intervals. Effect sizes whose confidence interval excludes zero are shown in white (positive) or black (negative); associations whose interval includes zero are shown in gray.

Unless otherwise stated, the associations reported below are Pearson correlations computed on the analytic sample of 103 women^1^. Descriptive statistics for every measured variable appear in Table 1, and the outcome of each prediction is summarized in Table 2 and shown in Figure 1.

**Table 1:** Means, standard deviations, and reliabilities of measured variables (*N* = 103).

Measure	Range	*M*	*SD*	*α*
Competitive altruism (total, 30 items)	1–5	2.51	0.78	0.95
Competitive altruism (women-directed, 15 items)	1–5	2.58	0.81	0.91
Competitive altruism (men-directed, 15 items)	1–5	2.43	0.88	0.92
Perceptions of other women’s CA toward women (10 items)	1–5	3.95	0.78	0.89
Perceptions of other women’s CA toward men (10 items)	1–5	4.03	0.65	0.84
Competitive altruism (manipulation, 14 items)	1–5	2.64	0.90	0.91
Competitive altruism (public-display, 14 items)	1–5	2.09	0.78	0.92
Competitive altruism (admiration-seeking, 4 items)	1–5	3.20	1.04	0.81
Anxiety-and-frustration composite (6 items)	1–5	2.54	0.97	0.85
Machiavellianism (SD3, 9 items)	1–5	2.92	0.71	0.81
Impression management (BIDR-IM, 20 items)	1–5	3.13	0.59	0.82
Marlowe–Crowne SDS (33 items, sum)	0–33	16.34	5.78	0.84
BFI-2 Extraversion (12 items)	1–5	3.01	0.80	0.87
BFI-2 Agreeableness (12 items)	1–5	3.84	0.65	0.85
BFI-2 Conscientiousness (12 items)	1–5	3.73	0.81	0.91
BFI-2 Neuroticism (12 items)	1–5	3.05	0.96	0.93
BFI-2 Openness (12 items)	1–5	3.87	0.73	0.88

CA = competitive altruism; BFI-2 = Big Five Inventory-2; BIDR-IM = Impression Management subscale of the Balanced Inventory of Desirable Responding; SDS = Social Desirability Scale; SD3 = Short Dark Triad. The Marlowe–Crowne SDS is scored as the sum of the 33 keyed responses (0–33). All other scale scores are computed as item means. Reliability values are Cronbach’s alpha.

**Table 2:** Confirmatory associations with competitive altruism and its composites (*N* = 103).

Hypothesis / association	*r*	95% CI	*p*	*BF*_10_	Outcome
H1a CA – Machiavellianism	+0.54	[0.39, 0.67]	<0.001^1^	> 1,000	Supported
H1b Mach – Manipulation	+0.57	[0.42, 0.69]	<0.001^1^	> 1,000	Supported
H1c Machiavellianism – Perceptions of other women’s CA	+0.24	[0.05, 0.42]	0.007^1^	2.5	Supported (women only)
H2a Agreeableness – Manipulation	−0.41	[−0.56, −0.24]	<0.001^1^	> 1,000	Supported
H2a Agreeableness – CA total	−0.36	[−0.52, −0.18]	<0.001	117.3	Null disconfirmed
H2b Extraversion – CA total	+0.02	[−0.18, 0.21]	0.866	0.12	Not significant
H2c Conscientiousness – CA total	−0.20	[−0.38, −0.004]	0.045	0.9	Not supported (direction reversed; BF inconclusive)
H2d Neuroticism – Anxiety + frustration	+0.36	[0.18, 0.52]	<0.001^1^	112.0	Supported
H2e Openness – CA total	−0.07	[−0.26, 0.12]	0.463	0.16	Not significant
H3a CA – Impression management	−0.46	[−0.60, −0.29]	<0.001	>1,000	Reversed
H3a CA – Social desirability	−0.36	[−0.52, −0.18]	<0.001	107.7	Reversed
H3b Impression management – Public display	−0.38	[−0.54, −0.20]	<0.001	300.1	Reversed
H3c Social desirability – Admiration items	−0.33	[−0.49, −0.15]	< 0.001	37.8	Specificity not supported
H4 CA women-directed > men-directed^2^	*d*_z_ + 0.27	[0.07, 0.46]^2^	0.008^1^	3.5	Supported

CA = competitive altruism, BF = Bayes factors. ^1^One-tailed, in the predicted direction; remaining p values are two-tailed. Bayes factors above 1,000 are reported as > 1,000. “Reversed” denotes a reliable association opposite to the registered prediction; “Null disconfirmed” denotes a reliable association where a negligible one was predicted. ^2^ For H4, the effect size shown in the r column is Cohen’s *d*_z_ and the 95% CI is on *d*_z_; H4 tests a paired mean difference between the eight-item audience subsets.

### Strategic self-presentation and social desirability

Competitive altruism correlated strongly and positively with Machiavellianism (*r* = 0.54, 95% CI [0.39, 0.67], one-tailed *p* < 0.001, *BF*_10_ > 1,000), supporting Hypothesis 1a by providing evidence that strategic kindness is part of a manipulative interpersonal orientation. After Benjamini–Hochberg correction the Machiavellianism correlation was the only one supported in the predicted direction. However, relationships with the two self-presentation measures ran opposite to prediction (Hypothesis 3), correlating negatively to impression management (*r* = −0.46, 95% CI [−0.60, −0.29], two-tailed *p* < 0.001, *BF*_10_ > 1,000) and to social desirability (*r* = −0.36, 95% CI [−0.52, −0.18], two-tailed *p* < 0.001, *BF*_10_ = 107.7). The negative relationships indicate that women who are more invested in managing impressions and in presenting themselves desirably report less competitive altruism rather than more.

### Machiavellianism, manipulation, and perceived competition

Hypothesis 1b was that Machiavellianism would correlate more strongly with the manipulation items of the CAS than with the rest of the scale. Machiavellianism was, as predicted, most clearly expressed in the manipulation items, correlating strongly with the manipulation composite (*r* = 0.57, 95% CI [0.42, 0.69], one-tailed *p* < 0.001, *BF*_10_ > 1,000). However, a Williams test against competitive altruism overall, the preregistered comparator, did not show specificity, *t*(100) = 0.96, *p* = 0.34, indicating that Machiavellianism is broadly associated with the scale rather than tied specifically to its manipulation items. Hypothesis 1c predicted that Machiavellianism would relate to the perception that other women use kindness strategically. This relationship was weak and carried by the same-sex audience, significant for perceptions of other women (*r* = 0.24, one-tailed *p* = 0.007) but not for perceptions of men (*r* = 0.09, one-tailed *p* = 0.173), with the Bayes factor for the overall perception association inconclusive (*BF*_10_ = 0.8).

### Impression management, approval seeking, and specificity

We predicted that the impression-management association with competitive altruism would be correlated most strongly with the public-display items (Hypothesis 3b), and that the social-desirability association would be correlated most strongly with the admiration and frustration items (Hypothesis 3c); these were not supported. Impression management related negatively to the public-display items (*r* = −0.38, 95% CI [−0.54, −0.20], two-tailed *p* < 0.001) rather than positively, and a Williams test against the manipulation items indicated that impression management was, if anything, more strongly but not significantly linked to the manipulation items than to the public-display items [*t*(100) = −1.68, *p* = 0.10]. Social desirability likewise correlated negatively to competitive altruism overall (*r* = −0.36) and to the admiration (*r* = −0.33, *BF*_10_ = 37.8) and frustration (*r* = −0.33, *BF*_10_ = 38.2) items, with no indication that the admiration or frustration items were more strongly linked than the total [Williams *t*(100) = 0.47, *p* = 0.64 for admiration; *t*(100) = 0.38, *p* = 0.71 for frustration].

### Personality correlates

Hypotheses 2a to 2e covered the Big Five Inventory-2, with directional predictions registered for Agreeableness (Hypothesis 2a, null on the total but negative on the manipulation items), Extraversion (2b, positive, particularly on the public-display items), Conscientiousness (2c, positive), and Neuroticism (2d, tied specifically to the anxiety-and-frustration composite), and no directional prediction for Openness (2e). Agreeableness correlated negatively to the manipulation items (*r* = −0.41, 95% CI [−0.56, −0.24], one-tailed *p* < 0.001, *BF*_10_ > 1,000), consistent with the dissociation of strategic kindness from warm prosociality, and Agreeableness correlated significantly negatively to the total (*r* = −0.36, two-tailed *p* < 0.001), with the Bayesian test providing strong evidence against the null (*BF*_10_ = 117.3). Extraversion was not significantly correlated to competitive altruism, with the Bayes factor favoring the null (*r* = 0.02, *BF*_10_ = 0.12, *BF*_01_ = 8.30), and showed no public-display effect. Conscientiousness was likewise not correlated in the predicted direction, and the Bayes factor was inconclusive (*r* = −0.20, *BF*_10_ = 0.9). Neuroticism, though, supported the hypothesis and was significantly correlated to the anxiety-and-frustration composite (*r* = 0.36, 95% CI [0.18, 0.52], one-tailed *p* < 0.001, *BF*_10_ = 112.0) and more so than to the remainder of the scale [*t*(100) = 2.47, *p* = 0.02], while the correlation with the competitive-altruism total was weak and the Bayes factor inconclusive (*r* = 0.20, *BF*_01_ = 1.10). Openness was insignificantly correlated to competitive altruism, with the Bayes factor favoring the null (*r* = −0.07, *BF*_10_ = 0.16, *BF*_01_ = 6.23).

### Audience effects

Hypothesis 4 predicted that competitive altruism would be more strongly reported for behaviors directed at other women than for behaviors directed at men. On the 8-item subsets of the women-directed and men-directed scales, women reported significantly more competitive altruism directed toward other women (*M* = 2.75, *SD* = 0.86) than toward men (*M* = 2.55, *SD* = 0.88), *t*(102) = 2.72, *p* = 0.008, *d*_z_ = 0.27, Wilcoxon *p* = 0.003. The same finding held on the full 15-item per-audience composites [women-directed *M* = 2.58, *SD* = 0.81; men-directed *M* = 2.43, *SD* = 0.88; *t*(102) = 2.25, *p* = 0.026]. However, the trait correlates of competitive altruism did not differ by audience for Machiavellianism [women audience *r* = 0.44, men audience *r* = 0.46, *t*(100) = −0.27, *p* = 0.790], or Agreeableness (women *r* = −0.25, men *r* = −0.28, *p* = 0.775). Only Extraversion showed a significant difference [women audience *r* = 0.11, men audience *r* = −0.07, *t*(100) = 2.11, *p* = 0.038], but the effect was small and rests on two near-zero correlations.

### Self-rated trait adjectives (exploratory)

The 13 peer-comparison self-ratings using adjectives were correlated with the CAS total, see Table 3. Women higher in competitive altruism rated themselves as significantly more manipulative, more deceptive, more fake, and less honest than other women their age. In contrast, there was no significant relationship between competitive altruism and self-rated kindness, generosity, or friendliness, meaning women who endorse strategic kindness do not see themselves as warmer than peers. Among the prestige-and-achievement adjectives, only Popular reached significance, with women scoring higher on the CAS rating themselves as more popular than other women their age, while Intelligent, Successful, Funny, Wealthy, and Ambitious were not significant. The pattern held when CAS was examined by audience sex, with the strategic adjectives and Popular correlating with the women and men audience composites to comparable degrees, and no clear audience specificity in self-perception. The Honest item yielded a negative correlation with competitive altruism that was significantly stronger when the audience was women than men, while the other strategic adjectives, Manipulative, Deceptive, and Fake, were evenly rated across the two audiences. Women who report directing strategic kindness specifically toward other women rate themselves as less honest than peers, whereas women whose strategic kindness is directed at men show no comparable pattern in self-reported honesty.

**Table 3:** Self-rated trait adjectives: means, standard deviations, and correlations with competitive altruism composites (*N* = 102–103).

Adjective	Cluster	*M*	*SD*	*r*	95% CI	*p*	*r* women-audience	*r* men-audience
Kind	Warmth	3.76	0.85	−0.12	[−0.30, 0.08]	0.233	−0.13	−0.09
Generous	Warmth	3.54	0.89	−0.07	[−0.26, 0.13]	0.502	−0.10	−0.03
Friendly	Warmth	3.53	0.96	+0.04	[−0.16, 0.23]	0.720	+0.08	−0.01
Intelligent	Prestige and achievement	3.72	0.77	+0.11	[−0.09, 0.30]	0.271	+0.07	+0.13
Successful	Prestige and achievement	2.71	1.10	+0.06	[−0.14, 0.25]	0.564	+0.01	+0.10
Popular	Prestige and achievement	2.40	1.00	+0.31	[0.12, 0.47]	0.001	+0.27	+0.30
Funny	Prestige and achievement	3.54	0.97	+0.08	[−0.12, 0.27]	0.445	−0.02	+0.15
Wealthy	Prestige and achievement	2.32	1.13	+0.16	[−0.04, 0.34]	0.108	+0.11	+0.18
Ambitious	Prestige and achievement	3.07	1.14	−0.01	[−0.20, 0.18]	0.905	−0.04	+0.02
Manipulative	Strategic and disagreeable	1.80	0.93	+0.37	[0.19, 0.52]	<0.001	+0.33	+0.35
Deceptive	Strategic and disagreeable	1.71	0.91	+0.33	[0.14, 0.49]	0.001	+0.23	+0.37
Fake	Strategic and disagreeable	1.53	0.80	+0.24	[0.05, 0.41]	0.015	+0.21	+0.23
Honest	Strategic and disagreeable (reverse)	4.01	0.82	−0.29	[−0.46, −0.10]	0.003	−0.35	−0.20

Items rated relative to other women of similar age on a five-point scale (1 = much less, 3 = about the same, 5 = much more). Total composite combines the 30 CAS items; women and men audience composites each comprise 15 items. *p*-values are two-tailed; no directional hypothesis was preregistered. Three adjectives (Generous, Friendly, Manipulative) are based on *N* = 102 due to a single missing response; remaining correlations use *N* = 103.

During review, it was suggested that we perform an exploratory check of whether competitive altruism differed between women who are and are not mothers. It did not significantly differ; women with children (*n* = 44, *M* = 2.48, *SD* = 0.91) and women without (*n* = 59, *M* = 2.53, *SD* = 0.68), Welch *t*(76.51) = −0.34, *p* = 0.74, Cohen’s *d* = −0.07. The survey did not record the ages of participants’ children, meaning an age-of-child analysis was not possible.

### Observed competitive altruism in other women

At the close of the survey, participants were invited to describe a time when they had noticed another woman using kindness or generosity to gain status or to manipulate others. Of the 103 women, 101 provided a response, and we summarize these findings here as a qualitative complement to the confirmatory findings and not as a formal test. The responses were independently coded by two trained coders, and the small number of discrepancies were resolved through discussion (Cohen’s κ = 0.63, 73%). One provisional category was found to conflate a setting with the tactics used within it, and as a result, the coding was refined to a single behavioral dimension, and the affected responses were resolved to consensus. Five recurring themes were identified, each describing the tactic a woman was seen to use. A single response could touch on more than one theme, and hence, the counts reflect the tactic judged most central to each response.

The most common theme, present in about a third of the responses (32 of 101), concerned generosity, in which a woman provided treats, food, gifts, or favors, to build goodwill. Respondents described a teacher who brought in candy for colleagues each week, a newcomer whose drinks were paid for so that she would be given a lift, and a colleague who used a gift or favor to place another person under obligation. The second theme, about a quarter of the responses (27 of 101), dealt with performed warmth, in which friendliness shown in public was contradicted in private or served a purpose rather than reflecting genuine regard. Respondents described a colleague who used pet names and was sickly sweet to gain favor, women who behaved as though they were friends with everyone in a workplace in a way that came across as insincere, and helpfulness before a promotion that was more staged than a genuine offer.

The third theme (15 of 101) was warmth directed at men, often in a mating-relevant context. Respondents described a woman being extra nice to a man so that he would buy her a drink, and warmth aimed at a man, or at those close to him, in order to be preferred. The fourth theme (14 of 101) concerned instrumental helpfulness, in which a woman offered effort or assistance, often to a superior, in order to gain advantage or obligation. Respondents described colleagues who were overly kind to managers in the hope of better assignments, and a junior staff member who took on menial tasks for senior figures to seem useful.

A fifth and smaller theme, 6 of 101, pertained to rivalry with other women, in which kindness was used to win standing among women or was paired with coldness toward female peers. Respondents described a woman who went above and beyond in her attention to a friend for a concealed motive, a friend who was sweet to people’s faces while speaking unkindly of them in their absence, and kindness offered to get closer to a woman for an ulterior end. The remaining 7 accounts were not related to any theme or were too vague to code. The settings participants described were overwhelmingly everyday and reputational, centered on workplaces, social groups, and online display, with open confrontation almost absent.

## Discussion

The present results qualify the conventional view of women as the more cooperative sex (e.g., Balliet et al., 2011; Eagly, 2009). While we agree that some of women’s cooperation is actually genuinely cooperative, we argue some is strategic, with a manipulative element, and it is mostly used by women whose interpersonal style is colder than the warmth of the behavior suggests. We created the Competitive Altruism Survey (CAS) to explore this possibility. Here, self-reported competitive altruism (measured by CAS) was strongly positively correlated with Machiavellianism and negatively correlated with the warm facets of personality (i.e., Agreeableness) and with impression management and social desirability. Women reported, in some instances, more competitive altruism when the audience was other women than men, and the open-ended responses indicated strategic kindness occurs in workplaces, social groups, and social media, and at times paired it with unkind talk about the same person in her absence. What remains to be determined is whether the strategic use of kindness succeeds in eliciting cooperation from observers. Indeed, the current research provides many directions for future work.

A concern is whether the CAS is distinct from Machiavellianism, given their strong correlation. However, the two differ in what they ask about. Machiavellianism, as measured by the Short Dark Triad (Jones and Paulhus, 2014), is a generalized cynicism about human nature and an instrumental and strategic orientation toward other people (Christie and Geis, 1970), without reference to any particular behavioral channel through which the orientation is expressed. The CAS specifies each item as an observable act of kindness, generosity, or helping that is reported as being used for strategic ends. The two measures should therefore be expected to correlate, since women who use kindness strategically also report a generally manipulative outlook, but they are not interchangeable.

The prediction that competitive altruism would be largely independent of Agreeableness was NOT supported, and the two were reliably and negatively related, with the manipulation items pulling the relationship. The open-ended responses provide further evidence of this pattern, as the kindness participants singled out as strategic was very often described as insincere or as a cover for unkindness, with warmth shown to a person’s face and criticism reserved for their absence. The trait correlates describe a calculating use of warmth, and not the eagerness to be liked that a conformist interpretation would predict. Genuine warmth earns prestige through actual care for others and should align with Agreeableness. Alternatively, feigned warmth, earns reputation through performance and need not involve Agreeableness at all, which is what was found. Reynolds et al. (2025) report the same asymmetry, as gossip phrased as concern damages its target’s reputation while leaving the gossiper looking more trustworthy and more desirable than baseline, which they proposed is evidence that a woman’s belief in her own benevolent motives is competitively useful. Strategically kind women may benefit for the same reason, given her kindness appears as genuine to observers and, very possibly, to the actor herself.

Extraversion was not significantly related to competitive altruism. The reputational reward that generosity is thought to bring in extraverted actors (Ashton et al., 2002; Hardy and Van Vugt, 2006) did not carry into the strategic use of kindness in this sample. The finding for Conscientiousness indicates strategic kindness is not a deliberate and planned pursuit. Openness was likewise not significant, suggesting curiosity has little influence. Neuroticism, in contrast, was significantly linked to the anxiety-and-frustration composite (but not competitive altruism overall). This finding is consistent with our hypothesis that the emotional cost of strategic kindness (i.e., the concern that one’s generosity might be perceived as insincere, the frustration of going unnoticed) is felt more by neurotic women even when strategic kindness is not.

Machiavellian women more readily attributed strategic kindness to other women than to men, with the same-sex association reaching significance, while the perception of men’s strategic kindness did not. What these findings suggest is that Machiavellian women watch other women more attentively for strategic use of kindness, and this result echoes the audience findings that women use competitive altruism more when the audience is women than men. Social desirability, in contrast, was reliably and negatively associated with competitive altruism. The negative association aligns with the possibility that women who most readily endorse strategic kindness are also least invested in socially desirable self-presentation. That is, a woman who deploys warmth strategically does not feel a need to be liked by the wider social audience.

Returning to the sex of audience members, the trait correlates of competitive altruism, Machiavellianism and low Agreeableness in particular, were of comparable size whether the behavior was directed at women or at men. This finding does not support our audience prediction, even though the mean-level component held, and the present correlations do not distinguish a disposition specific to same-sex audiences from a more general one. The pattern is consistent with a wider account of women’s social lives, in which much of the cooperative work, much of the rivalry, and much of the reputational management have always been conducted in the company of other women (Hrdy, 2009), so that the audience for strategic kindness will tend to be female by default. More research on this area is needed to determine the full influence of sex of those who receive the competitive altruism, as well as other variables such as their age, reproductive status and so on.

Earlier research on competitive altruism has focused on men and conspicuous public display, with male generosity functioning as a costly signal addressed to potential mates (Iredale et al., 2008; McAndrew and Perilloux, 2012; Raihani and Smith, 2015). The present findings point to a relational, often female-directed counterpart in women, deployed within workplaces, friendship groups, and online life rather than as a broadcast display. The qualitative responses include publicized generosity of the kind observed in men, indicating the contrast is one of degree. We identify a complementary form of competitive altruism but do not claim that it is exclusive to women; further research is needed.

Competitive altruism also needs to be distinguished from the constructs with which it overlaps. Manipulation, self-promotion, impression management, and approval seeking each capture part of what the survey assesses, yet none are interchangeable with the survey. A desire for admiration may be a motive for competitive altruism, conspicuous display as a tactic through which it operates, and frustration at unnoticed kindness a consequence. Competitive altruism is the strategic use of prosocial behavior for advantage, while the adjacent constructs name the motives, tactics, and feelings that accompany it (Hardy and Van Vugt, 2006; Simpson and Willer, 2008). More research may be advantageous to determine how these issues interrelate.

Do women who use strategic kindness actually admit to its use, or do they hide it as part of their strategy? One possible answer is simply that the CAS taps into women’s willingness to admit strategic kindness rather than deny its use; the key here being the openness to admission. There may be differences in self-presentation style, or a greater willingness among women high in Machiavellianism and low in Agreeableness to endorse socially undesirable items. The trait-adjective self-ratings show that women higher in competitive altruism describe themselves, by direct peer comparison, as more manipulative, deceptive, and fake than other women, and not as warmer. The problem, though, is that strategically kind women who are unwilling to admit their motivations will score low on the scale and adjectives, meaning it will underestimate the behavior precisely among the women most invested in appearing kind. The reliance on self-report is an issue, given self-deception can facilitate the deception of others, so an implicit or reaction-time measure of strategically motivated kindness would be a useful complement to the present design and perhaps could begin to address this problem.

Another limitation is that we do not know whether the women who score highly on the CAS are extended greater cooperation, alliance, or status by those they are kind to, or if any such returns are larger when kindness is directed at other women than at men. Settling that question will require designs that observe both the actor and the recipient. Public-goods games with reputation manipulations, in which participants’ generosity is made variably visible to subsequent cooperative partners, would test directly whether competitive altruism translates into higher returns when the audience is watching (Hardy and Van Vugt, 2006). Partner-choice paradigms, in which participants select cooperative partners from candidates who differ in observable generosity, would establish whether women high in competitive altruism are preferentially chosen and by whom (Barclay and Willer, 2007). Observational coding of dyads or small groups would extend the test to less artificial settings, with kindness coded as it occurs and recipients’ subsequent cooperation, alliance, or resource-sharing recorded as outcomes. Across all three approaches, manipulating the sex of the audience would be especially important for the present question, given that competitive altruism in women is hypothesized to operate most strongly within same-sex contexts.

While the CAS showed high internal consistency, it is a newly developed scale and the findings must be treated as tentative. Further studies are required to examine its factor structure in larger, more diverse samples, assess test–retest reliability, and establish validity relative to other variables. In particular, future studies should investigate whether CAS scores predict visible reputational behavior, mate choice, alliance formation, or subsequent cooperation, beyond general Machiavellianism. An act-nomination procedure would also help anchor a revised version of the scale to behaviors specifically, rather than to feelings or perceptions. Further, the open-ended responses, while adding useful information, concern women’s perceptions of others rather than their own behavior, so the converging evidence remains within the self-report frame. The sample, of heterosexual cisgender women drawn predominantly from the United Kingdom, the United States, and Canada, is modest and not highly representative, and the cross-sectional design cannot speak to direction of effect or to within-person change over time. Indeed, the generalizability of these findings is bounded culturally. Kindness, indirect competition, self-presentation, reputational hierarchy, and judgments of sincerity are culturally embedded (e.g., Henrich et al., 2010). The norms governing acceptable self-promotion and female status competition may vary across settings, so the predominantly Western, English-speaking composition of the sample limits the reach of the conclusions, and cross-cultural replication with measurement-invariance testing would be a valuable next step.

Although the current study is preliminary, it provides an important first look into women’s competitive altruism as a coherent psychological construct. Higher CAS scores relate with Machiavellianism and low Agreeableness rather than warmth, and women who endorse the strategic use of kindness rate themselves as more manipulative, deceptive, and popular than their peers. Kindness is offered in order to be seen offering it and directed more often at other women than at men, aligning with an intrasexual competitive use of indirect aggression. The reversed correlations with impression management and social desirability clearly indicates competitive altruism is not just self-presentation or approval seeking. Replication in larger and more culturally varied samples is one next step, as is longitudinal work testing whether the audience effect shifts with reproductive context, and behavioral measures to complement the self-report scales.

## Data Availability

The preregistration, anonymized data, and analysis materials for this study are available on the Open Science Framework at https://osf.io/78pve/. Further enquiries can be directed to the corresponding author.

## Footnotes

1 The restriction of the analytic sample to heterosexual participants was not preregistered. A sensitivity analysis including the two participants excluded for identifying as non-heterosexual (*N* = 105) left every result minimally changed, with competitive altruism correlating *r* = 0.55 with Machiavellianism, *r* = −0.46 with impression management, *r* = −0.36 with social desirability, and *r* = −0.37 with Agreeableness, and with the audience difference remaining significant, *t*(104) = 2.84, *p* = 0.005.

## References

[ref1] Ashton M. C. Lee K. Paunonen S. V. (2002). What is the central feature of extraversion? Social attention versus reward sensitivity. J. Pers. Soc. Psychol. 83, 245–252. doi: 10.1037/0022-3514.83.1.245, 12088129

[ref2] Balliet D. Li N. P. Macfarlan S. J. Van Vugt M. (2011). Sex differences in cooperation: a meta-analytic review of social dilemmas. Psychol. Bull. 137, 881–909. doi: 10.1037/a0025354, 21910518

[ref3] Barclay P. (2013). Strategies for cooperation in biological markets, especially for humans. Evol. Hum. Behav. 34, 164–175. doi: 10.1016/j.evolhumbehav.2013.02.002

[ref4] Barclay P. Willer R. (2007). Partner choice creates competitive altruism in humans. Proc. R. Soc. B Biol. Sci. 274, 749–753. doi: 10.1098/rspb.2006.0209, 17255001 PMC2197220

[ref5] Batson C. D. (2011). Altruism in Humans. New York: Oxford University Press.

[ref6] Benenson J. F. (2013). The development of human female competition: allies and adversaries. Phil. Trans. R. Soc. B 368:20130079. doi: 10.1098/rstb.2013.0079, 24167309 PMC3826208

[ref7] Benenson J. F. Abadzi H. (2020). Contest versus scramble competition: sex differences in the quest for status. Curr. Opin. Psychol. 33, 62–68. doi: 10.1016/j.copsyc.2019.07.013, 31400660

[ref8] Benjamini Y. Hochberg Y. (1995). Controlling the false discovery rate: a practical and powerful approach to multiple testing. J. R. Statistic. Soc. 57, 289–300. doi: 10.1111/j.2517-6161.1995.tb02031.x

[ref9] Böhm R. Regner T. (2013). Charitable giving among females and males: an empirical test of the competitive altruism hypothesis. J. Bioecon. 15, 251–267. doi: 10.1007/s10818-013-9152-x

[ref10] Campbell A. (1999). Staying alive: evolution, culture, and women’s intrasexual aggression. Behav. Brain Sci. 22, 203–214. doi: 10.1017/S0140525X99001818, 11301523

[ref11] Cassar A. Rigdon M. L. (2021). Prosocial option increases women’s entry into competition. Proc. Natl. Acad. Sci. 118:e2111943118. doi: 10.1073/pnas.2111943118, 34725163 PMC8609315

[ref12] Cassar A. Wordofa F. Zhang Y. J. (2016). Competing for the benefit of offspring eliminates the gender gap in competitiveness. Proc. Natl. Acad. Sci. 113, 5201–5205. doi: 10.1073/pnas.1520235113, 27114513 PMC4868415

[ref13] Christie R. Geis F. L. (1970). Studies in Machiavellianism. New York: Academic Press.

[ref14] Crowne D. P. Marlowe D. (1960). A new scale of social desirability independent of psychopathology. J. Consult. Psychol. 24, 349–354. doi: 10.1037/h0047358, 13813058

[ref15] David-Barrett T. Rotkirch A. Carney J. Behncke Izquierdo I. Krems J. A. Townley D. . (2015). Women favour dyadic relationships, but men prefer clubs: cross-cultural evidence from social networking. PLoS One 10:e0118329. doi: 10.1371/journal.pone.0118329, 25775258 PMC4361571

[ref16] Eagly A. H. (2009). The his and hers of prosocial behavior: an examination of the social psychology of gender. Am. Psychol. 64, 644–658. doi: 10.1037/0003-066X.64.8.644, 19899859

[ref17] Fehr E. Fischbacher U. (2003). The nature of human altruism. Nature 425, 785–791. doi: 10.1038/nature02043, 14574401

[ref18] Fisher M. L. (Ed.). (2017). The Oxford Handbook of Women and Competition. New York: Oxford University Press.

[ref19] Fisher M. L. (2026). The battle of the brides: co-wife competition and aggression across 19 cultures. Evol. Hum. Behav. 47:106957. doi: 10.1016/j.evolhumbehav.2026.106957

[ref20] Fisher M. Cox A. (2011). Four strategies used during intrasexual competition for mates. Pers. Relat. 18, 20–38. doi: 10.1111/j.1475-6811.2010.01307.x

[ref21] Giardini F. Vilone D. Sánchez A. Antonioni A. (2021). Gossip and competitive altruism support cooperation in a public good game. Phil. Trans. R. Soc. B Biol. Sci. 376:20200303. doi: 10.1098/rstb.2020.0303, 34601909 PMC8487737

[ref22] Habashi M. M. Graziano W. G. Hoover A. E. (2016). Searching for the prosocial personality: a big five approach to linking personality and prosocial behavior. Personal. Soc. Psychol. Bull. 42, 1177–1192. doi: 10.1177/0146167216652859 27401167

[ref23] Hardy C. L. Van Vugt M. (2006). Nice guys finish first: the competitive altruism hypothesis. Personal. Soc. Psychol. Bull. 32, 1402–1413. doi: 10.1177/0146167206291006, 16963610

[ref24] Henrich J. Heine S. J. Norenzayan A. (2010). The weirdest people in the world? Behav. Brain Sci. 33, 61–83. doi: 10.1017/S0140525X0999152X, 20550733

[ref25] Hrdy S. B. (2009). Mothers and others: The Evolutionary Origins of Mutual Understanding. Cambridge, MA: Belknap Press of Harvard University Press.

[ref26] Iredale W. Jenner K. Van Vugt M. Dempster T. (2020). Giving guys get the girls: men appear more desirable to the opposite sex when displaying costly donations to the homeless. Soc. Sci. 9:141. doi: 10.3390/socsci9080141

[ref27] Iredale W. Van Vugt M. Dunbar R. (2008). Showing off in humans: male generosity as a mating signal. Evol. Psychol. 6, 386–392. doi: 10.1177/147470490800600302

[ref28] Jones D. N. Paulhus D. L. (2014). Introducing the short dark triad (SD3): a brief measure of dark personality traits. Assessment 21, 28–41. doi: 10.1177/1073191113514105, 24322012

[ref29] Leary M. R. Kowalski R. M. (1990). Impression management: a literature review and two-component model. Psychol. Bull. 107, 34–47. doi: 10.1037/0033-2909.107.1.34

[ref30] McAndrew F. T. Perilloux C. (2012). Is self-sacrificial competitive altruism primarily a male activity? Evol. Psychol. 10, 50–65. doi: 10.1177/147470491201000107, 22833848 PMC10480791

[ref31] Nowak M. A. (2006). Five rules for the evolution of cooperation. Science 314, 1560–1563. doi: 10.1126/science.1133755, 17158317 PMC3279745

[ref32] Paulhus D. L. (1984). Two-component models of socially desirable responding. J. Pers. Soc. Psychol. 46, 598–609. doi: 10.1037/0022-3514.46.3.598

[ref33] Paulhus D. L. (1991). “Measurement and control of response bias,” in Measures of Personality and Social Psychological Attitudes, eds. Robinson J. P. Shaver P. R. Wrightsman L. S. (San Diego: Academic Press), 17–59.

[ref34] Raihani N. J. Smith S. (2015). Competitive helping in online giving. Curr. Biol. 25, 1183–1186. doi: 10.1016/j.cub.2015.02.042, 25891407

[ref35] Reynolds T. A. Maner J. K. Baumeister R. F. (2025). Bless her heart: gossip phrased with concern provides advantages in female intrasexual competition. J. Exp. Soc. Psychol. 116:104670. doi: 10.1016/j.jesp.2024.104670

[ref36] Roberts G. (1998). Competitive altruism: from reciprocity to the handicap principle. Proc. R. Soc. B Biol. Sci. 265, 427–431. doi: 10.1098/rspb.1998.0312

[ref37] Rucas S. L. Gurven M. Kaplan H. Winking J. Gangestad S. Crespo M. (2006). Female intrasexual competition and reputational effects on attractiveness among the Tsimane of Bolivia. Evol. Hum. Behav. 27, 40–52. doi: 10.1016/j.evolhumbehav.2005.07.001

[ref38] Simpson B. Willer R. (2008). Altruism and indirect reciprocity: the interaction of person and situation in prosocial behavior. Soc. Psychol. Q. 71, 37–52. doi: 10.1177/019027250807100106

[ref39] Soto C. J. John O. P. (2017). The next big five inventory (BFI-2): developing and assessing a hierarchical model with 15 facets to enhance bandwidth, fidelity, and predictive power. J. Pers. Soc. Psychol. 113, 117–143. doi: 10.1037/pspp0000096, 27055049

[ref40] Sylwester K. Roberts G. (2010). Cooperators benefit through reputation-based partner choice in economic games. Biol. Lett. 6, 659–662. doi: 10.1098/rsbl.2010.0209, 20410026 PMC2936156

[ref41] Tognetti A. Dubois D. Faurie C. Willinger M. (2016). Men increase contributions to a public good when under sexual competition. Sci. Rep. 6:29819. doi: 10.1038/srep29819, 27412070 PMC4944141

[ref42] Trivers R. L. (1971). The evolution of reciprocal altruism. Phil. Trans. R. Soc. B 46, 35–57. doi: 10.1086/406755

[ref43] Vaillancourt T. (2013). Do human females use indirect aggression as an intrasexual competition strategy? Philos. Transact. R. Soc. B 368:20130080. doi: 10.1098/rstb.2013.0080, 24167310 PMC3826209

[ref44] Van Vugt M. Roberts G. Hardy C. (2007). “Competitive altruism: a theory of reputation-based cooperation in groups,” in Oxford Handbook of Evolutionary Psychology, eds. Dunbar R. I. M. Barrett L. (Oxford: Oxford University Press), 531–540.

[ref45] Webb A. Fisher M. L. (2018). Distribution of resources to panhandlers as a male display of potential mate quality. Hum. Ethol. Bull. 33, 28–36. doi: 10.22330/heb/334/028-036

[ref46] West S. A. Griffin A. S. Gardner A. (2007). Evolutionary explanations for cooperation. Curr. Biol. 17, R661–R672. doi: 10.1016/j.cub.2007.06.004, 17714660

[ref47] Williams E. J. (1959). The comparison of regression variables. J. R. Statistic. Soc. 21, 396–399. doi: 10.1111/j.2517-6161.1959.tb00346.x

[ref48] Zahavi A. (1975). Mate selection—a selection for a handicap. J. Theor. Biol. 53, 205–214. doi: 10.1016/0022-5193(75)90111-3, 1195756

