# Supplementary material for: Kill them with kindness: preliminary evidence for women’s competitive altruism
Source: Front Psychol. 2026 Sep 9;17:1924479. doi: 10.3389/fpsyg.2026.1924479 (PMC13597864; doi:10.3389/fpsyg.2026.1924479)
Supplement: Supplementary file 1 [file Supplementary_file_1.docx]

**Appendix A**

**Competitive Altruism Survey (CAS)**

All items below were rated on a five-point agreement scale ranging from 1 (strongly disagree) to 5 (strongly agree). Items are listed in the order in which they were presented to participants.

**Competitive Altruism Behaviors — directed at other women (15 items)**

1. I often go out of my way to appear kind or generous in front of other women.

2. I have done favors for other women to gain their admiration or approval.

3. I sometimes use acts of kindness to gain an advantage in social or professional settings with other women.

4. I feel the need to outshine other women by being more helpful or generous.

5. I have exaggerated my generosity to make other women think highly of me.

6. I have volunteered for tasks or events to appear more selfless than other women.

7. I have used compliments or flattery to gain favor with other women.

8. I have posted about my acts of kindness or generosity on social media to gain attention from other women.

9. I have helped another woman with the intention of receiving something in return.

10. I feel competitive with other women when it comes to being seen as kind or generous.

11. I have used kindness or generosity to gain favor with a romantic partner’s female friends or family members.

12. I have acted overly nice to another woman I don’t like to avoid conflict or gain her trust.

13. I have felt satisfaction when other women noticed my acts of kindness or generosity.

14. I have felt frustrated when my acts of kindness or generosity went unnoticed by other women.

15. I have tried to appear more generous or selfless than I actually am in front of other women.

**Competitive Altruism Behaviors — directed at men (15 items)**

1. I often go out of my way to appear kind or generous in front of men.

2. I have done favors for men to gain their admiration or approval.

3. I sometimes use acts of kindness to gain an advantage in social or professional settings with men.

4. I feel the need to outshine other women by being more helpful or generous in front of men.

5. I have exaggerated my generosity to make men think highly of me.

6. I have volunteered for tasks or events to appear more selfless in front of men.

7. I have used compliments or flattery to gain favor with men.

8. I have posted about my acts of kindness or generosity on social media to gain attention from men.

9. I have helped a man with the intention of receiving something in return.

10. I feel competitive with other women when it comes to being seen as kind or generous in front of men.

11. I have used kindness or generosity to gain favor with a romantic partner or his male friends or family members.

12. I have acted overly nice to a man I don’t like to avoid conflict or gain his trust.

13. I have felt satisfaction when men noticed my acts of kindness or generosity.

14. I have felt frustrated when my acts of kindness or generosity went unnoticed by men.

15. I have tried to appear more generous or selfless than I actually am in front of men.

**Perceptions of Competitive Altruism in Other Women — toward other women (10 items)**

1. I have noticed women competing with other women to appear more kind or generous.

2. I believe some women use kindness or generosity to manipulate other women.

3. I think women sometimes act overly nice to gain social status or popularity among other women.

4. I have seen women use acts of kindness to gain favor in professional settings with other women.

5. I believe some women use generosity as a way to outshine other women in social groups.

6. I think women are more likely than men to use kindness or generosity competitively with other women.

7. I feel uncomfortable when another woman’s kindness toward other women seems insincere or performative.

8. I believe social media encourages women to exaggerate their acts of kindness or generosity to impress other women.

9. I think women are more likely to act overly kind or generous to gain recognition or status in environments where other women are present, such as workplaces or schools.

10. I believe that when women act overly kind or generous toward other women for personal gain, it can harm relationships by creating distrust.

**Perceptions of Competitive Altruism in Other Women — toward men (10 items)**

1. I have noticed women competing to appear more kind or generous in front of men.

2. I believe some women use kindness or generosity to manipulate men.

3. I think women sometimes act overly nice to gain social status or popularity among men.

4. I have seen women use acts of kindness to gain favor in professional settings with men.

5. I believe some women use generosity as a way to outshine other women in front of men.

6. I think women are more likely than men to use kindness or generosity competitively to gain the attention or approval of men.

7. I feel uncomfortable when a woman’s kindness toward men seems insincere or performative.

8. I believe social media encourages women to exaggerate their acts of kindness or generosity to impress men.

9. I think women are more likely to act overly kind or generous to gain recognition or status in environments where men are present, such as workplaces or schools.

10. I believe that when women act overly kind or generous toward men for personal gain, it can harm relationships by creating distrust.

**Self-Reflection Items (7 items)**

1. I feel good about myself when I help others, even if no one notices.

2. I sometimes feel the need to prove my kindness or generosity to other women.

3. I sometimes feel the need to prove my kindness or generosity in front of men.

4. I have felt competitive with other women in social or professional settings.

5. I believe my acts of kindness or generosity are always genuine.

6. I sometimes worry that other women perceive my kindness as insincere.

7. I sometimes worry that men perceive my kindness as insincere.

**Competitive Altruism Survey: subset assignments**

| **Statement (rated once for women, once for men)** | **Manipulation** | **Public display** | **Admiration** |
| --- | --- | --- | --- |
| 1. …appear kind or generous in front of… |  | ✓ |  |
| 2. …done favors… to gain admiration or approval |  |  | ✓ |
| 3. …use acts of kindness to gain an advantage… | ✓ |  |  |
| 4. …outshine… by being more helpful or generous |  | ✓ |  |
| 5. …exaggerated generosity to make… think highly of me | ✓ | ✓ |  |
| 6. …volunteered… to appear more selfless |  | ✓ |  |
| 7. …used compliments or flattery to gain favor | ✓ |  |  |
| 8. …posted… on social media to gain attention |  | ✓ |  |
| 9. …helped… with the intention of receiving something in return | ✓ |  |  |
| 10. …feel competitive… about being seen as kind or generous |  | ✓ |  |
| 11. …gain favor with a romantic partner’s friends or family | ✓ |  |  |
| 12. …acted overly nice to someone I do not like… | ✓ |  |  |
| 13. …felt satisfaction when… noticed my acts of kindness |  |  | ✓ |
| 14. …felt frustrated when my acts… went unnoticed |  |  |  |
| 15. …tried to appear more generous or selfless than I actually am | ✓ | ✓ |  |

Note. Each statement was rated once with respect to other women and once with respect to men, so each tick indicates two rated items and the subsets comprise 14, 14, and 4 items respectively. Statements 5 and 15 contribute to both the manipulation and public-display subsets. Statement 14 belongs to none of the three subsets; it forms part of the separate anxiety-and-frustration composite described in the Method.

The preregistration additionally specified an eight-item index for each audience. The women-directed index comprised statements 1, 2, 4, 5, 6, 7, 13 and 14, and the men-directed index statements 1, 2, 3, 4, 8, 11, 12 and 13. This defines the ‘8-item subsets’ referred to in the Results (Audience effects) and in footnote 2 to Table 2.

**Principal component loadings**

| **Statement** | **Toward women** | **Toward men** |
| --- | --- | --- |
| 1. …appear kind or generous in front of… | .35 | .57 |
| 2. …done favors… to gain admiration or approval | .68 | .67 |
| 3. …use acts of kindness to gain an advantage… | .70 | .71 |
| 4. …outshine… by being more helpful or generous | .68 | .73 |
| 5. …exaggerated generosity to make… think highly of me | .72 | .81 |
| 6. …volunteered… to appear more selfless | .61 | .70 |
| 7. …used compliments or flattery to gain favor | .64 | .63 |
| 8. …posted… on social media to gain attention | .45 | .49 |
| 9. …helped… with the intention of receiving something in return | .64 | .56 |
| 10. …feel competitive… about being seen as kind or generous | .66 | .73 |
| 11. …gain favor with a romantic partner’s friends or family | .53 | .53 |
| 12. …acted overly nice to someone I do not like… | .51 | .46 |
| 13. …felt satisfaction when… noticed my acts of kindness | .59 | .63 |
| 14. …felt frustrated when my acts… went unnoticed | .67 | .62 |
| 15. …tried to appear more generous or selfless than I actually am | .72 | .83 |

Note. Loadings on the first unrotated principal component, which accounted for 40.5% of the item variance, for each statement rated with respect to other women and with respect to men.
